# Antibiotic producing microorganisms from River Wiwi, Lake Bosomtwe and the Gulf of Guinea at Doakor Sea Beach, Ghana

**DOI:** 10.1186/1471-2180-12-234

**Published:** 2012-10-16

**Authors:** Adelaide A Tawiah, Stephen Y Gbedema, Francis Adu, Vivian E Boamah, Kofi Annan

**Affiliations:** 1 Department of Pharmaceutics, Faculty of Pharmacy and Pharmaceutical Sciences, College of Health Sciences, Kwame Nkrumah University of Science and technology, Kumasi, Ghana; 2 Department of Pharmacognosy, Faculty of Pharmacy and Pharmaceutical Sciences, College of Health Sciences, Kwame Nkrumah University of Science and technology, Kumasi, Ghana

**Keywords:** Aquatic microorganisms, Antibiotics, Ghana, Multi-drug resistance

## Abstract

**Background:**

Microorganisms have provided a wealth of metabolites with interesting activities such as antimicrobial, antiviral and anticancer. In this study, a total of 119 aquatic microbial isolates from 30 samples (taken from water bodies in Ghana) were screened by the agar-well diffusion method for ability to produce antibacterial-metabolites.

**Results:**

Antibacterial activity was exhibited by 27 of the isolates (14 bacteria, 9 actinomycetes and 4 fungi) against at least one of the indicator microorganisms: *Enterococcus faecalis* (ATCC 29212)*, Bacillus thuringiensis* (ATCC 13838)*, Pseudomonas aeruginosa* (ATCC 27853)*, Staphylococcus aureus* (ATCC 25923)*, Proteus vulgaris* (NCTC 4635) *and Bacillus Subtilis* (NCTC 10073). A sea isolate MAI2 (identified as a strain of *Pseudomonas aeruginosa*) exhibited the highest antibacterial activity (lowest zone of inhibition = 22 mm). The metabolites of MAI2 extracted with chloroform were stable to heat and gave minimum inhibitory concentrations ranging between 250 and 2000 μg/ml. Bioautography of the extract revealed seven active components.

**Conclusion:**

This study has therefore uncovered the potential of water bodies in the West African sub-region as reservoirs of potent bioactive metabolite producing microorganisms.

## Background

Throughout the ages, natural products have been the most consistently successful source of lead compounds that have found many applications in the fields of medicine, pharmacy and agriculture. Microbial natural products have been the source of most of the antibiotics in current use for the treatment of various infectious diseases. Since the discovery of penicillin in 1928, studies on soil bacteria and fungi have shown that microorganisms are a rich source of structurally unique bioactive substances [1]. After Penicillin, many other drugs including chlortetracycline, chloramphenicol, streptomycin, erythromycin, rifamycin, lincomycin, cephalosporin C, vancomycin, erythromycin, nalidixic acid, amphotericin B, nystatin, and daunorubicin the antitumor agent were discovered from microorganisms. Currently, many of the pathogens implicated in infectious disease are rapidly developing resistance to the available antibiotics [2] making treatment of these infections very difficult [3], hence the need to look for more effective antibiotics.

Until recently, majority of antimicrobial compounds were isolated from terrestrial microorganisms. In the last two decades however, the rate of discovery of novel compounds from this source has significantly declined, as exemplified by the fact that extracts from soil-derived actinomycetes have yielded high numbers of clinically unacceptable metabolites [4]. The aquatic environment is now becoming increasingly appreciated as a rich and untapped reservoir of useful novel natural products. The marine environment alone is known to contain taxonomically diverse bacterial groups which exhibit unique physiological and structural characteristics that enable them to survive in extreme environmental conditions, with the potential production of novel secondary metabolites not observed in terrestrial microorganisms [5]. Several compounds including pestalone, hypoxysordarin and equisetin, isolated from sea microorganisms have shown promising antibacterial, antifungal and antiviral activities respectively. Salinosporamide A isolated from marine *Salinispora tropica,* has been shown to exhibit both anticancer and antimalarial activities and is currently undergoing clinical trial [6].

In Ghana and other sub-Saharan African countries is a diverse array of aquatic habitats. These water bodies are reservoirs of enormous biological diversity which have not been exploited for bioactive natural products. In this study therefore, we report the presence of potent antimicrobial metabolite producing microorganisms in some aquatic habitats in Ghana.

## Methods

### Sampling and Isolation of microorganisms

The Gulf of Guinea at Cape Coast Duakor Sea beach and two fresh water bodies in the Ashanti region of Ghana; Lake Bosomtwe and River Wiwi, were selected for this study. Thirty samples of water, weeds, stones and sediments were collected from each of these sites and transported at 4°C to the laboratory. Water samples were collected by submerging sterile 1 L glass bottles in the water to a depth of about 10 cm and then opened to fill after which they were closed and brought to surface. About five grams (5 g) each of sediment materials, stones and weed in the water bodies were collected into bottles.

All samples were processed within 12 hours of collection. About 1 ml quantities of the water samples were separately inoculated into 20 ml molten Nutrient agars and Sabouraud agars (Merck, Nottingham, UK). The stones and weed samples were gently and separately scrubbed with sterile brush into10 ml sterile normal saline and 1 ml quantities were added to the molten agars. About 1 g of the soil samples were also suspended in 5 ml of normal saline and 1 ml of these suspensions were added to the agars. All the plates were incubated (Nutrient agars at 37°C and Sabouraud agars at 25°C) for seven days with daily observation. Colonies that appeared to have clear zones around them were carefully isolated into pure cultures.

### Test microorganisms

These microorganisms from the stocks kept by the Microbiology Laboratory of the Department of Pharmaceutics were used in the study: *Bacillus thuringiensis* (ATCC 13838), *Staphylococcus aureus* (ATCC 25923), *Bacillus subtilis* (NCTC 10073), *Pseudomonas aeruginosa* (ATCC 27853), *Proteus vulgaris* (NCTC 4175), *Enterococcus faecalis* (ATCC 29212), *Escherichia coli* (clinical isolate), *Salmonella typhi* (clinical isolate) and *Candida albicans* (clinical isolate).

### Screening of isolated microorganisms for inhibitory activity

The isolates were screened for antibacterial metabolite production using the agar-well diffusion method. The inocula were prepared by growing the various test organisms on separate agar plates and colonies from the plate were transferred with inoculating loop into 3 ml of normal saline in a test tube. The density of these suspensions was adjusted to 0.5 McFarland standards. The surface of Muller-Hinton agar (Oxoid Cambridge, UK) plate was evenly inoculated with the test organisms using a sterile swab: the swab was dipped into the suspension and pressed against the side of the test tube to remove excess fluid. The wet swab was then used to inoculate the Muller-Hinton agar by evenly streaking across the surface. By means of a sterile cork borer wells (8 mm in diameter) were made in the agar and filled with 0.2 ml of 72 h culture of the isolate microorganism. Two replicates of the experiment were done and the plates incubated at 37°C for 18 h. The diameters of zone of growth-inhibition produced were measured and the mean values calculated (Table 1). Isolates MAI1, MAI2 and MAI3 produced the highest zones and were therefore selected for the next level of studies.

**Table 1:** **Antimicrobial activity of isolatesagainst the test microorganismsemployed**

**Producers**		**Mean zones of growth-inhibition(mm) of test organisms(±SEM)**					
		**PA**	**BT**	**BS**	**EF**	**SA**	**PV**
SAI 19	Ac	14 ± 1.12	16 ± 0.11	15 ± 0.41	11 ± 0.21	14 ± 2.0	15 ± 0.21
SAI 22	Ac	-	-	11 ± 3.05	14 ± 2.22	11 ± 0.07	12 ± 1.20
SAI 20	Br	-	11 ± 0.66	-	11 ± 0.02	-	13 ± 0.10
SAI 28	Br	-	12 ± 2.12	-	13 ± 0.01	-	11 ± 2.07
SAI 29	Ac	-	14 ± 0.31	13 ± 0.77	14 ± 0.73	-	-
SAI 18	Br	-	12 ± 1.11	-	12 ± 1.27	-	12 ± 1.16
SAI 9	Br	-	10 ± 1.54	-	-	-	-
SAI 12	Br	-	12 ± 0.97	-	-	-	12 ± 0.16
SAI 36	Ac	-	13 ± 0.76	13 ± 0.76	14 ± 0.46	14 ± 1.17	12 ± 0.55
SAI 31	Ac	-	12 ± 3.27	-	11 ± 3.09	-	-
SAI 32	Fg	-	12 ± 0.09	11 ± 0.83	12 ± 2.39	13 ± 0.09	12 ± 1.43
SAI 35	Br	-	14 ± 0.04	14 ± 0.98	14 ± 4.01	12 ± 2.17	12 ± 2.44
SAI 23	Br	-	-	-	-	-	12 ± 0.26
SAI 5	Fg	-	-	11 ± 0.45	-	-	11 ± 0.15
WEI 3	Ac	-	14 ± 1.22	14 ± 0.11	15 ± 1.44	15 ± 0.11	13 ± 0.03
WEI 7	Br	-	11 ± 4.11	-	12 ± 0.33	12 ± 0.43	-
WEI 13	Fg	-	11 ± 0.23	-	13 ± 0.76	-	11 ± 3.27
WEI 14	Ac	-	14 ± 2.91	13 ± 3.23	16 ± 1.28	13 ± 4.30	13 ± 1.30
WEI 16	Br	-	-	-	11 ± 2.99	-	-
WEI 19	Br	-	-	-	10 ± 1.19	-	-
BS 1	Ac	13 ± 4.09	14 ± 5.10	15 ± 1.22	12 ± 0.61	13 ± 2.99	14 ± 0.91
BS 8	Br	-	-	-	-	-	17 ± 2.07
BS 26	Fg	-	-	13 ± 0.22	15 ± 0.09	-	-
MAI 1	Br	-	20 ± 0.11	17 ± 0.26	22 ± 1.40	20 ± 0.18	17 ± 0.99
MAI 2	Br	-	24 ± 1.16	26 ± 2.33	22 ± 2.14	-	25 ± 3.17
MAI 3	Br	-	-	20 ± 2.19	22 ± 0.49	-	-
MAI 4	Ac	-	-	-	15 ± 0.87	-	-

Key: *Ac* = Actinomycetes, *Br* = Bacteria, *Fg* = fungi, *PA* = *P. aeruginosa*, *EF* = *E. faecalis*, *BT* = *B. thuringensis*, *SA* = *Staph aureus*, *BS* = *B. Subtilis*, *PV* = *Pr.* vulgaris. *SAI* = Sand isolates from River Wiwi, *WEI* = weed isolates from River Wiwi, *MAI* = marine isolates, *BS* = isolates from Lake Bosomtwe.

### Testing thermal stability of antibacterial metabolites of selected isolates

About 1 ml of the broth cultures of isolates MAI1, MAI2 and MAI3 were separately inoculated into 10 ml nutrient broths and incubated at 37°C for 72 hours. They were then centrifuged at 6000 rpm for one hour to precipitate the microbial cells from the metabolite solutions. The resulting supernatants were decanted and filtered through Whatman (No. 1) filter paper into clean sterile test tubes in 1 ml quantities and exposed to various temperatures from 40 to 121°C for 15 min. They were then re-tested for antimicrobial activity against *B. subtilis*. The metabolites of MAI2 showed better stability and hence was finally selected for further studies.

### Effect of growth factors on antibacterial activity of MAI2 metabolites

#### Incubation period

The incubation period for maximum activity of MAI2 was assessed by fermenting it in 250 ml of nutrient broth in a shaking incubator at 37°C. Aliquots of 10 ml of the culture were withdrawn at 24 h intervals and centrifuged as above. The cell-free supernatant was assayed for inhibitory activity against *B. subtilis*.

*pH*: The optimum pH for maximum activity was assessed by fermenting MAI2 in tubes of 10 ml nutrient broth at varying pHs (4, 5, 6, 7, 8 and 9) after which their antibacterial activity was evaluated by the cup plate method as above.

#### Carbon and nitrogen sources

The source of carbon for optimum activity was assessed by cultivating the isolate in 10 ml fermentation media fortified with 60 mg of various carbon sources: glucose, galactose, xylose, sucrose, mannitol, lactose, starch, fructose, maltose and glycerol. The metabolite solutions obtained were tested for antimicrobial activity against *B. subtilis.* The procedure was repeated for nitrogen sources (asparagine, sodium nitrate, potassium nitrate, ammonium chloride, ammonium nitrate, ammonium phosphate and ammonium sulphate).

### Extraction of metabolites of Isolate MAI2

The isolate was inoculated into 2.5 L of nutrient broth and incubated at 37°C for 10 days. The culture was then centrifuged at 6000 rpm for 1 h and the supernatant filtered, extracted with chloroform and dried at room temperature (25°C). Two replicates were done and the extracts obtained were weighed and kept in a desiccator for use.

### Minimum inhibitory and bactericidal concentrations determination of MAI2 extract

Minimum Inhibitory Concentration (MIC) was determined using the broth dilution method. Serial dilutions (100 μl) of the extract in Mueller-Hinton Broth (Sigma-Aldrich, St. Louis, MO, USA) in the range of 62.5 μg/ml to 4000 μg/ml were made in 96-well micro-plates. The inocula (100 μl) of the test microorganisms prepared from 18 h broth cultures (containing 10^5^ cfu/ml) were dispensed into the plates. Three replicates were made. The plates were incubated at 37°C for 24 hours. Bacterial growth was determined after addition of 20 μl of 0.2 mg/ml MTT (Sigma-Aldrich, St. Louis, MO, USA).

The minimum bactericidal concentration (MBC) test was performed as above in the MIC determination except that 100 μl aliquots were withdrawn from wells that showed inhibition in the MIC experiment and inoculated into 5 ml nutrient broths. These were incubated at 37°C for 5 days and observed for signs of growth.

### Bioautography assay

**Figure 1:**
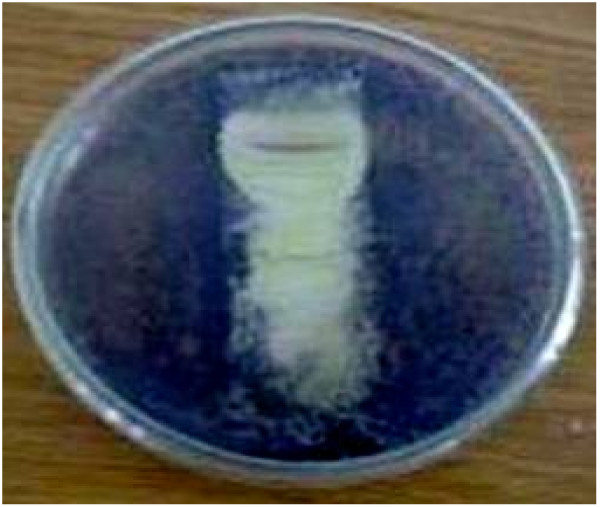
** Bioautography of MAI2 extract against *Pr.vulgaris*.**

Bioautography as described by Nostro *et al.*[7] was performed using *Pr. vulgaris* which showed a good sensitivity to the crude extracts. Briefly, developed and dried Silica gel 60 microns TLC plates (Merck, Nottingham, UK) were overlaid with agar seeded with an overnight culture of *Pr. vulgaris*. The plates were incubated for 24 h at 37°C and then sprayed with an aqueous solution of 2 mg/ml MTT. Zones of growth inhibition appeared clear against a purple background (Figure 1). 

### Characterization of isolate MAI2

The morphological features of the colonies including sizes, shapes, colour and pigmentation and microscopic features of the cells in addition to biochemical tests such as growth on cetrimide agar, indole and oxidase production, citrate utilization, starch hydrolysis and carbohydrate fermentations were used to characterize isolate MAI2 in accordance with Barrow and Felthan [8]. *Pseudomonas aeruginosa* (ATCC 27853) was employed as the reference organism. The other 26 active isolates were as well identified and classified as bacteria, actinomycetes or fungi.

## Results

### Isolation, antibacterial activity and thermal stability

**Figure 2:**
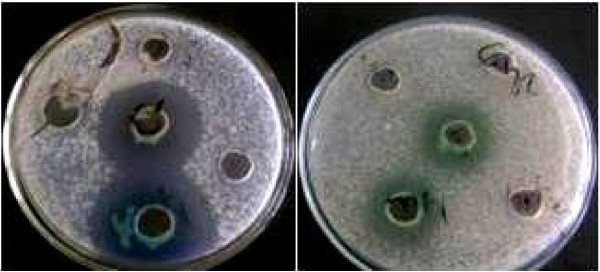
** Samples of the agar plates showing zones of growth inhibition.**

A total of 119 isolates suspected of having the capability to produce inhibitory metabolites were recovered from the 30 samples collected, out of which 27 (23%) (made up of 14 bacteria, 9 actinomycetes and 4 fungi) actually exhibited antimicrobial properties (determined by zone of growth inhibition ≥ 10 mm) against at least one of the test bacteria used (Figure 2; Table 1). 66.7% of the strains inhibited *B. thuringiensis*, 60% inhibited *B. subtilis*, 37% inhibited *Staph. aureus*, 66.7% inhibited *Pr. vulgaris* and 81.48% inhibited *Ent. faecalis*. Only two of the isolates inhibited *P. aeruginosa*. Three of the bacterial isolates (MAI1, MAI2 and MAI3) produced inhibition zones greater than 19 mm but their antibacterial activity was lost on exposure to temperatures beyond 60°C except MAI2 which maintained activity up to 100°C. As such MAI2 was selected for further evaluation of its antibiotic and also identified to be a strain of *P. aeruginosa*.

**Figure 3:**
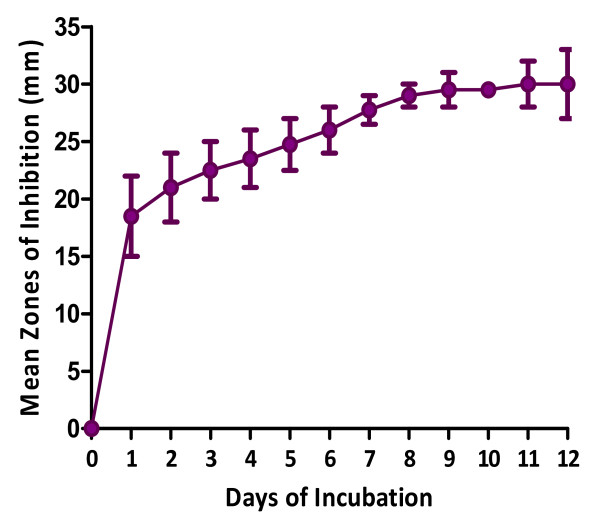
** Incubation period and antibacterial activity of MAI2 against *B. Subtilis*.**

**Figure 4:**
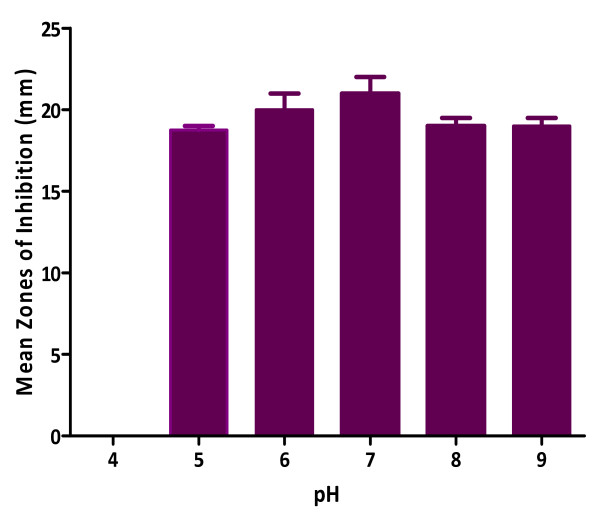
** Effect of pH on antibacterial activity of Isolate MAI2.**

**Figure 5:**
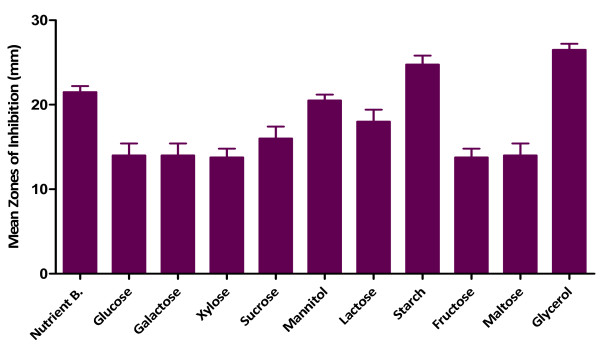
** Effect of carbon sources on antimicrobial activity of MAI2 against *B. subtilis*.**

**Figure 6:**
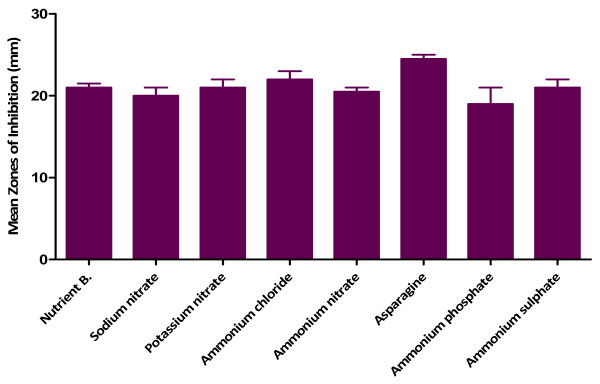
** Effect of nitrogen sources on antibacterial activity of MAI2 against *B. Subtilis*.**

There was an increase in the antibacterial activity of MAI2 metabolites up to the ninth day of incubation after which there was no significant increase (p < 0.005; Figure 3). The optimum pH for maximum antibacterial activity of MAI2 was determined to be 7 and no activity was observed at pH of 4 (Figure 4). Fortification of the fermentation medium with glycerol produced the highest activity followed by starch as carbon sources (Figure 5) while asparagine gave the highest activity in the case of nitrogen sources (Figure 6). The effects of all the other carbon and nitrogen sources were either equal or significantly lower than the control (nutrient broth).

### Extraction and antimicrobial activity of crude extract

The crude extract obtained (0.281 g per 2.5 L fermentation medium) was dark brown in colour and exhibited activity against *E. coli*, *Pr. vulgaris*, *Ent. faecalis*, *Staph. aureus*, *B. subtilis, B. thuringiensis*, *S. typhi* and *C. albicans* with MIC values ranging between 250 to 2000 μg/ml (Table 2). Growth was however observed in all the tubes in the MBC determination at the concentrations tested.

**Table 2:** **MIC of the crudeextract of MAI2**

**Test organism**	**MIC in μg/ml**
*E. coli*	500
*Pr. vulgaris*	250
*Ent. Faecalis*	500
*Staph. aureus*	1000
*B. subtilis*	250
*B. thuringiensis*	1000
*S. typhi*	500
*C. albicans*	2000

### TLC analysis

TLC of the crude extract showed 7 components under UV light at 254 nm and the R_f_ values of the spots are 0.86, 0.77, 0.55, 0.52, 0.44, 0.30 and 0.22 in chloroform-ethyl acetate (3.5:1.5) solvent system. All the components appeared to have exhibited antibacterial activity (Figure 1).

## Discussion

New and effective antibiotics are crucial in this current surge of multi-drug resistant bacterial infections which have rendered many of the currently available antibiotics useless. Natural products have served and continue to provide useful lead compounds for development into chemotherapeutic agents. Aquatic microorganisms have emerged as a source of diverse chemical compounds which have not been adequately studied for chemotherapeutic application. Our results have revealed 27 (23%) antibiotic producing microorganism out of 119 isolates recovered from both marine and fresh water sources in Ghana and this is the first report of this kind of study in the West African sub-region. Many reports have been made of such studies elsewhere. For example Ivanova *et al*. [9] reported that out of the 491 bacteria isolated from different marine sources, 26% of the isolates were active. Zheng *et al*. [10] also reported that 8 out of 29 strains, representing 28% of the isolates considered in their study produced antimicrobial activity against at least one of their test microorganisms. Brandelli *et al*. [11] also recorded 70% of active isolates from the Amazon Basin whilst O’Brien *et al*. [12] recorded as low as 0.29% (13 out of 4496) of active microbes from soil samples collected at different location in the Antarctica.

The comparatively high number of antibiotic producers recorded in our study can be partly attributed to the nature of our water bodies: they are usually highly polluted with all kinds of waste materials; from domestic and industrial wastewater discharges, mining runoff, agro-chemicals and other sources [13-16] and river wiwi, Lake Bosomtwe and the Gulf of Guinea at Duakor Sea Beach where the samples were collected are no exceptions. To survive and maintain their niche under these harsh conditions therefore, the aquatic microorganisms need defense mechanisms and for some, antimicrobially active metabolite production could be one of such mechanisms. The differences among the detection rates reported in literature strongly depend on the isolation and assay procedures, test organisms, type of media used, as well as the sources of bacterial isolates [17]. In our study, only those isolates producing extracellular antibiotics were detected, hence very huge numbers could be recorded if our procedures include microorganisms producing intracellular antibiotics since they will only secrete their antibiotics into media in the presence of competition, to antagonise other organisms for survival [18].

Isolate MAI2 which was identified as a strain of *Pseudomonas aeruginosa*, exhibited the highest antibacterial activity and produced perhaps, moderately thermo-stable antibacterial metabolites, shown by exhibition of antibacterial activity when the metabolites solution was exposed to temperatures up to 100°C but destroyed at 121°C for 15 min. This result is in contrast to those reported by Preetha *et al.*[19] who reported that the antimicrobial agent produced by *Pseudomonas* species MCCB was stable after autoclaving at 121°C for 20 min even though there was a significant reduction in activity. Uzair *et al*. [20] also reported the thermal stability of an antimicrobial agent produced by *Pseudomonas aeruginosa* at a temperature of 121°C for 20 minutes. However, Roitman *et al*. [21] showed that variations in the fermentation medium often results in changes in the composition of the antibiotics produced. The differences in the thermal stability of the antimicrobial agents produced in this study as compared to other studies may therefore be due to differences in some nutritional and or physical factors which led to the production of metabolites that are thermolabile at temperatures beyond 100°C.

Our results also showed that nine days incubation period was optimum for maximum antibacterial activity by MAI2, an indication of maximum antibiotic production, after which there was no significant increase. Several other factors influence production of secondary metabolites by microorganisms, the most important one being the composition of the fermentation medium [22]. Sole *et al*. [23] noted that glucose can be used as a source for bacterial growth while repressing the production of secondary metabolites. The isolate (MAI2) utilised glycerol and starch best for maximum production of the antimicrobial metabolites.

Nitrogen is very vital in the synthesis of enzymes involved in primary and secondary metabolism [24]. Therefore depending on the biosynthetic pathways involved, nitrogen sources may affect antibiotic formation. Shapiro [25] noted that the type of nitrogen source (organic or inorganic) plays a role in the synthesis of secondary metabolites. Charyulu and Gnanamani [26] reported that *Pseudomonas aeruginosa* MTCC 5210 utilized organic nitrogen source for better yield of antimicrobial metabolites than the inorganic sources. These observations are consistent with the findings of this study as asparagine was better used for antibiotic production by MAI2 than the inorganic nitrogen sources (sodium and potassium nitrates and the ammonium salts) employed.

Generally, the intracellular pH of most microorganisms is maintained near neutrality regardless of the pH in the outside medium [27]. However as the proton gradient across the cytoplasmic membrane increases, the cells commit more of their resources towards maintaining the desired intracellular pH [28], thus changes in external pH affect many cellular processes such as growth and the regulation of the biosynthesis of secondary metabolites [29]. The highest activity of the antimicrobial metabolite by the strain was at pH 7. This result agrees with a study carried out by Charyulu and Gnanamani [26] who reported maximum production of metabolite by *Pseudomonas aeruginosa* MTCC 5210 at pH 7.

Isolate MAI2 exhibited antimicrobial activity against both gram-positive and gram-negative microorganisms as well as *C. albicans*, indicating that the metabolites have a broad antimicrobial spectrum.

The seven components observed in the TLC analysis of the extract points to the fact that organisms can produce more than one antimicrobial agent to provide themselves with survival competition superiority. Further work is ongoing in our laboratory to isolate and test the various components of the extract. It is hoped that these components when isolated into pure constituents can serve as leads for the development of novel and potent antibiotics as well as resistant reversing compounds [30,31] which may be useful in combination therapies as exemplified by clavulanic acid in Augmentin^R^ (Glaxo-SmithKline).

The extract is bacteriostatic in its mode of action since there were revivable cells of the test organisms in the wells in which inhibition was observed. Bacteriostatic agents like the β- lactams have been of great value in the treatment of bacterial infections including endocarditis, meningitis, and osteomyelitis [32]. Other bacteriostatic agents such as the lincosamides (example clindamycin) have been shown to completely inhibit the toxic shock syndrome toxin-1 production by *Staph. aureus*[33] and toxin production in both streptococci and staphylococci [34]. These reports suggest that the active constituents MAI2 crude extract have the potential of being efficacious in the treatment of various infections.

## Conclusions

It was found out from this study that antibiotic producing microorganisms are present in Lake Bosomtwe, river wiwi at KNUST campus and the Gulf of Guinea at Duakor Sea beach. Out of the 119 isolates recovered, 27 produced antibacterial metabolites against at least one of the test organisms. The crude metabolite extract of isolate MAI2 (a strain of *P. aeruginosa*) was active against all the test organisms; *B. thuringiensis*, *Pr. vulgaris*, *Ent. faecalis*, *Staph. aureus*, *B. subtilis*, *E. coli, S. typhi* and *C. albicans* with MICs ranging between 250 and 2000 μg/ml.

## Competing interests

The authors declare that they have no competing interest.

## Authors’ contributions

SYG conceived and designed the experimental plan, AAT performed most of the experiments, FA and KA performed chromatographic analysis, SYG, AAT and VEB analysed data and wrote the manuscript; all authors have reviewed the manuscript. All authors read and approved the final manuscript.
